# The persistence of pay inequality: The gender pay gap in an anonymous online labor market

**DOI:** 10.1371/journal.pone.0229383

**Published:** 2020-02-21

**Authors:** Leib Litman, Jonathan Robinson, Zohn Rosen, Cheskie Rosenzweig, Joshua Waxman, Lisa M. Bates

**Affiliations:** 1 Department of Psychology, Lander College, Flushing, New York, United States of America; 2 Department of Computer Science, Lander College, Flushing, New York, United States of America; 3 Department of Health Policy & Management, Mailman School of Public Health, Columbia University, New York, New York, United States of America; 4 Department of Clinical Psychology, Columbia University, New York, New York, United States of America; 5 Department of Computer Science, Stern College for Women, New York, New York, United States of America; 6 Department of Epidemiology, Mailman School of Public Health, Columbia University New York, New York, United States of America; Northwestern University, UNITED STATES

## Abstract

Studies of the gender pay gap are seldom able to simultaneously account for the range of alternative putative mechanisms underlying it. Using CloudResearch, an online microtask platform connecting employers to workers who perform research-related tasks, we examine whether gender pay discrepancies are still evident in a labor market characterized by anonymity, relatively homogeneous work, and flexibility. For 22,271 Mechanical Turk workers who participated in nearly 5 million tasks, we analyze hourly earnings by gender, controlling for key covariates which have been shown previously to lead to differential pay for men and women. On average, women’s hourly earnings were 10.5% lower than men’s. Several factors contributed to the gender pay gap, including the tendency for women to select tasks that have a lower advertised hourly pay. This study provides evidence that gender pay gaps can arise despite the absence of overt discrimination, labor segregation, and inflexible work arrangements, even after experience, education, and other human capital factors are controlled for. Findings highlight the need to examine other possible causes of the gender pay gap. Potential strategies for reducing the pay gap on online labor markets are also discussed.

## Introduction

The gender pay gap, the disparity in earnings between male and female workers, has been the focus of empirical research in the US for decades, as well as legislative and executive action under the Obama administration [1, 2]. Trends dating back to the 1960s show a long period in which women’s earnings were approximately 60% of their male counterparts, followed by increases in women’s earnings starting in the 1980s, which began to narrow, but not close, the gap which persists today [3]. More recent data from 2014 show that overall, the median weekly earnings of women working full time were 79–83% of what men earned [4–9].

The extensive literature seeking to explain the gender pay gap and its trajectory over time in traditional labor markets suggests it is a function of multiple structural and individual-level processes that reflect both the near-term and cumulative effects of gender relations and roles over the life course. Broadly speaking, the drivers of the gender pay gap can be categorized as: 1) human capital or productivity factors such as education, skills, and workforce experience; 2) industry or occupational segregation, which some estimates suggest accounts for approximately half of the pay gap; 3) gender-specific temporal flexibility constraints which can affect promotions and remuneration; and finally, 4) gender discrimination operating in hiring, promotion, task assignment, and/or compensation. The latter mechanism is often estimated by inference as a function of unexplained residual effects of gender on payment after accounting for other factors, an approach which is most persuasive in studies of narrowly restricted populations of workers such as lawyers [10] and academics of specific disciplines [11]. A recent estimate suggests this unexplained gender difference in earnings can account for approximately 40% of the pay gap [3]. However, more direct estimations of discriminatory processes are also available from experimental evidence, including field audit and lab-based studies [12–14]. Finally, gender pay gaps have also been attributed to differential discrimination encountered by men and women on the basis of parental status, often known as the ‘motherhood penalty’ [15].

### Non-traditional ‘gig economy’ labor markets and the gender pay gap

In recent years there has been a dramatic rise in nontraditional ‘gig economy’ labor markets, which entail independent workers hired for single projects or tasks often on a short-term basis with minimal contractual engagement. “Microtask” platforms such as Amazon Mechanical Turk (MTurk) and Crowdflower have become a major sector of the gig economy, offering a source of easily accessible supplementary income through performance of small tasks online at a time and place convenient to the worker. Available tasks can range from categorizing receipts to transcription and proofreading services, and are posted online by the prospective employer. Workers registered with the platform then elect to perform the advertised tasks and receive compensation upon completion of satisfactory work [16]. An estimated 0.4% of US adults are currently receiving income from such platforms each month [17], and microtask work is a growing sector of the service economy in the United States [18]. Although still relatively small, these emerging labor market environments provide a unique opportunity to investigate the gender pay gap in ways not possible within traditional labor markets, due to features (described below) that allow researchers to simultaneously account for multiple putative mechanisms thought to underlie the pay gap.

The present study utilizes the Amazon Mechanical Turk (MTurk) platform as a case study to examine whether a gender pay gap remains evident when the main causes of the pay gap identified in the literature do not apply or can be accounted for in a single investigation. MTurk is an online microtask platform that connects employers (‘requesters’) to employees (‘workers’) who perform jobs called “Human Intelligence Tasks” (HITs). The platform allows requesters to post tasks on a dashboard with a short description of the HIT, the compensation being offered, and the time the HIT is expected to take. When complete, the requester either approves or rejects the work based on quality. If approved, payment is quickly accessible to workers. The gender of workers who complete these HITs is not known to the requesters, but was accessible to researchers for the present study (along with other sociodemographic information and pay rates) based on metadata collected through CloudResearch (formerly TurkPrime), a platform commonly used to conduct social and behavioral research on MTurk [19].

Evaluating pay rates of workers on MTurk requires estimating the pay per hour of each task that a worker accepts which can then be averaged together. All HITs posted on MTurk through CloudResearch display how much a HIT pays and an estimated time that it takes for that HIT to be completed. Workers use this information to determine what the corresponding hourly pay rate of a task is likely to be, and much of our analysis of the gender pay gap is based on this advertised pay rate of all completed surveys. We also calculate an estimate of the gender pay gap based on actual completion times to examine potential differences in task completion speed, which we refer to as estimated actual wages (see Methods section for details).

Previous studies have found that both task completion time and the selection of tasks influences the gender pay gap in at least some gig economy markets. For example, a gender pay gap was observed among Uber drivers, with men consistently earning higher pay than women [20]. Some of the contributing factors to this pay gap include that male Uber drivers selected different tasks than female drivers, including being more willing to work at night and to work in neighborhoods that were perceived to be more dangerous. Male drivers were also likely to drive faster than their female counterparts. These findings show that person-level factors like task selection, and speed can influence the gender pay gap within gig economy markets.

MTurk is uniquely suited to examine the gender pay gap because it is possible to account simultaneously for multiple structural and individual-level factors that have been shown to produce pay gaps. These include discrimination, work heterogeneity (leading to occupational segregation), and job flexibility, as well as human capital factors such as experience and education.

#### Discrimination

When employers post their HITs on MTurk they have no way of knowing the demographic characteristics of the workers who accept those tasks, including their gender. While MTurk allows for selective recruitment of specific demographic groups, the MTurk tasks examined in this study are exclusively open to all workers, independent of their gender or other demographic characteristics. Therefore, features of the worker’s identity that might be the basis for discrimination cannot factor into an employer’s decision-making regarding hiring or pay.

#### Task heterogeneity

Another factor making MTurk uniquely suited for the examination of the gender pay gap is the relative homogeneity of tasks performed by the workers, minimizing the potential influence of gender differences in the type of work pursued on earnings and the pay gap. Work on the MTurk platform consists mostly of short tasks such as 10–15 minute surveys and categorization tasks. In addition, the only information that workers have available to them to choose tasks, other than pay, is the tasks’ titles and descriptions. We additionally classified tasks based on similarity and accounted for possible task heterogeneity effects in our analyses.

#### Job flexibility

MTurk is not characterized by the same inflexibilities as are often encountered in traditional labor markets. Workers can work at any time of the day or day of the week. This increased flexibility may be expected to provide more opportunities for participation in this labor market for those who are otherwise constrained by family or other obligations.

#### Human capital factors

It is possible that the more experienced workers could learn over time how to identify higher paying tasks by virtue of, for example, identifying qualities of tasks that can be completed more quickly than the advertised required time estimate. Further, if experience is correlated with gender, it could contribute to a gender pay gap and thus needs to be controlled for. Using CloudResearch metadata, we are able to account for experience on the platform. Additionally, we account for multiple sociodemographic variables, including age, marital status, parental status, education, income (from all sources), and race using the sociodemographic data available through CloudResearch.

### Expected gender pay gap findings on MTurk

Due to the aforementioned factors that are unique to the MTurk marketplace–e.g., anonymity, self-selection into tasks, relative homogeneity of the tasks performed, and flexible work scheduling–we did not expect a gender pay gap to be evident on the platform to the same extent as in traditional labor markets. However, potential gender differences in task selection and completion speed, which have implications for earnings, merit further consideration. For example, though we expect the relative homogeneity of the MTurk tasks to minimize gender differences in task selection that could mimic occupational segregation, we do account for potential subtle residual differences in tasks that could differentially attract male and female workers and indirectly lead to pay differentials if those tasks that are preferentially selected by men pay a higher rate. To do this we categorize all tasks based on their descriptions using K-clustering and add the clusters as covariates to our models. In addition, we separately examine the gender pay gap within each topic-cluster.

In addition, if workers who are experienced on the platform are better able to find higher paying HITs, and if experience is correlated with gender, it may lead to gender differences in earnings. Theoretically, other factors that may vary with gender could also influence task selection. Previous studies of the pay gap in traditional markets indicate that reservation wages, defined as the pay threshold at which a person is willing to accept work, may be lower among women with children compared to women without, and to that of men as well [21]. Thus, if women on MTurk are more likely to have young children than men, they may be more willing to accept available work even if it pays relatively poorly. Other factors such as income, education level, and age may similarly influence reservation wages if they are associated with opportunities to find work outside of microtask platforms. To the extent that these demographics correlate with gender they may give rise to a gender pay gap. Therefore we consider age, experience on MTurk, education, income, marital status, and parental status as covariates in our models.

Task completion speed may vary by gender for several reasons, including potential gender differences in past experience on the platform. We examine the estimated actual pay gap per hour based on HIT payment and estimated actual completion time to examine the effects of completion speed on the wage gap. We also examine the gender pay gap based on advertised pay rates, which are not dependent on completion speed and more directly measure how gender differences in task selection can lead to a pay gap. Below, we explain how these were calculated based on meta-data from CloudResearch.

To summarize, the overall goal of the present study was to explore whether gender pay differentials arise within a unique, non-traditional and anonymous online labor market, where known drivers of the gender pay gap either do not apply or can be accounted for statistically.

## Materials and methods

### Data

#### Amazon mechanical turk and CloudResearch

Started in 2005, the original purpose of the Amazon Mechanical Turk (MTurk) platform was to allow requesters to crowdsource tasks that could not easily be handled by existing technological solutions such as receipt copying, image categorization, and website testing. As of 2010, researchers increasingly began using MTurk for a wide variety of research tasks in the social, behavioral, and medical sciences, and it is currently used by thousands of academic researchers across hundreds of academic departments [22]. These research-related HITs are typically listed on the platform in generic terms such as, “Ten-minute social science study,” or “A study about public opinion attitudes.”

Because MTurk was not originally designed solely for research purposes, its interface is not optimized for some scientific applications. For this reason, third party add-on toolkits have been created that offer critical research tools for scientific use. One such platform, CloudResearch (formerly TurkPrime), allows requesters to manage multiple research functions, such as applying sampling criteria and facilitating longitudinal studies, through a link to their MTurk account. CloudResearch’s functionality has been described extensively elsewhere [19]. While the demographic characteristics of workers are not available to MTurk requesters, we were able to retroactively identify the gender and other demographic characteristics of workers through the CloudResearch platform. CloudResearch also facilitates access to data for each HIT, including pay, estimated length, and title.

The study was an analysis of previously collected metadata, which were analyzed anonymously. We complied with the terms of service for all data collected from CloudResearch, and MTurk. The approving institutional review board for this study was IntegReview.

#### Analytic sample

We analyzed the nearly 5 million tasks completed during an 18-month period between January 2016 and June 2017 by 12,312 female and 9,959 male workers who had complete data on key demographic characteristics. To be included in the analysis a HIT had to be fully completed, not just accepted, by the worker, and had to be accepted (paid for) by the requester. Although the vast majority of HITs were open to both males and females, a small percentage of HITs are intended for a specific gender. Because our goal was to exclusively analyze HITs for which the requesters did not know the gender of workers, we excluded any HITs using gender-specific inclusion or exclusion criteria from the analyses. In addition, we removed from the analysis any HITs that were part of follow-up studies in which it would be possible for the requester to know the gender of the worker from the prior data collection. Finally, where possible, CloudResearch tracks demographic information on workers across multiple HITs over time. To minimize misclassification of gender, we excluded the 0.3% of assignments for which gender was unknown with at least 95% consistency across HITs.

#### Measures

The main exposure variable is worker gender and the outcome variables are estimated *actual* hourly pay accrued through completing HITs, and *advertised* hourly pay for completed HITs. Estimated actual hourly wages are based on the estimated length in minutes and compensation in dollars per HIT as posted on the dashboard by the requester. We refer to actual pay as estimated because sometimes people work multiple assignments at the same time (which is allowed on the platform), or may simultaneously perform other unrelated activities and therefore not work on the HIT the entire time the task is open. We also considered several covariates to approximate human capital factors that could potentially influence earnings on this platform, including marital status, education, household income, number of children, race/ethnicity, age, and experience (number of HITs previously completed). Additional covariates included task length, task cluster (see below), and the serial order with which workers accepted the HIT in order to account for potential differences in HIT acceptance speed that may relate to the pay gap.

### Analysis

#### Database and analytic approach

Data were exported from CloudResearch’s database into Stata in long-form format to represent each task on a single row. For the purposes of this paper, we use “HIT” and “study” interchangeably to refer to a study put up on the MTurk dashboard which aims to collect data from multiple participants. A HIT or study consist of multiple “assignments” which is a single task completed by a single participant. Columns represented variables such as demographic information, payment, and estimated HIT length. Column variables also included unique IDs for workers, HITs (a single study posted by a requester), and requesters, allowing for a multi-level modeling analytic approach with assignments nested within workers. Individual assignments (a single task completed by a single worker) were the unit of analysis for all models.

Linear regression models were used to calculate the gender pay gap using two dependent variables 1) women’s estimated actual earnings relative to men’s and 2) women’s selection of tasks based on advertised earnings relative to men’s. We first examined the actual pay model, to see the gender pay gap when including an estimate of task completion speed, and then adjusted this model for advertised hourly pay to determine if and to what extent a propensity for men to select more remunerative tasks was evident and driving any observed gender pay gap. We additionally ran separate models using women’s advertised earnings relative to men’s as the dependent variable to examine task selection effects more directly. The fully adjusted models controlled for the human capital-related covariates, excluding household income and education which were balanced across genders. These models also tested for interactions between gender and each of the covariates by adding individual interaction terms to the adjusted model. To control for within-worker clustering, Huber-White standard error corrections were used in all models.

#### Cluster analysis

To explore the potential influence of any residual task heterogeneity and gender preference for specific task type as the cause of the gender pay gap, we use K-means clustering analysis (seed = 0) to categorize the types of tasks into clusters based on the descriptions that workers use to choose the tasks they perform. We excluded from this clustering any tasks which contained certain gendered words (such as “male”, “female”, etc.) and any tasks which had fewer than 30 respondents. We stripped out all punctuation, symbols and digits from the titles, so as to remove any reference to estimated compensation or duration. The features we clustered on were the presence or absence of 5,140 distinct words that appeared across all titles. We then present the distribution of tasks across these clusters as well as average pay by gender and the gender pay gap within each cluster.

## Results

The demographics of the analytic sample are presented in Table 1. Men and women completed comparable numbers of tasks during the study period; 2,396,978 (48.6%) for men and 2,539,229 (51.4%) for women.

**Table 1:** Demographic characteristics of analytic sample.

	Men	Women
Total	N = 9959 (45%)	N = 12312 (55%)
**Total HITs**	N = 2,396,978 (48.6%)	N = 2,539,229 (51.4%)
**HITs per worker, Mean**	241	206
**Age, Mean (SD)**	36.0 (11.3)	38.4 (12.0)
**Income (%)**		
< 20K	25.0	25.5
20–39K	27.9	29.4
40–59K	22.9	21.5
60K+	24.2	23.6
**Children, Mean (SD)**	.46 (.65)	.74 (.73)
**Marital Status (%)**		
Never Married	55.2	36.7
Currently Married	37.9	49.0
Previously Married	7.0	4.2
**Education (%)**		
No college	52.0	55.2
College	36.3	33.0
Post college	11.7	11.8
**Race (%)**		
White	76.6	76.4
Asian	6.4	8.1
Black	10.4	7.5
Hispanic	4.7	5.3
Other	1.9	2.7

In Table 2 we measure the differences in remuneration between genders, and then decompose any observed pay gap into task completion speed, task selection, and then demographic and structural factors. Model 1 shows the unadjusted regression model of gender differences in estimated actual pay, and indicates that, on average, tasks completed by women paid 60 (10.5%) cents less per hour compared to tasks completed by men (t = 17.4, p < .0001), with the mean estimated actual pay across genders being $5.70 per hour.

**Table 2:** Linear regression of estimated actual hourly pay on gender (N = 4,936,207).

**Estimated Actual Pay** **Model 1:** Predictor: Gender Adjustment: None	**β**	**SE**	**95% CI**	**p-value**

Women	-0.60	.04	-0.69, -0.51	< .0001
**Model 2:** Predictor: Gender Adjustment: Advertised hourly pay				
Women	-0.46	.04	-0.55, -0.38	< .0001
**Model 3:** Predictor: Gender Adjustment: Advertised hourly pay + covariates*				
Women	-0.32	.05	-0.42, -0.23	< .0001

*Model adjusted for race, marital status, number of children and task clusters as categorical covariates, and age, HIT acceptance speed, and number of HITs as continuous covariates.

In Model 2, adjusting for advertised hourly pay, the gender pay gap dropped to 46 cents indicating that 14 cents of the pay gap is attributable to gender differences in the selection of tasks (t = 8.6, p < .0001). Finally, after the inclusion of covariates and their interactions in Model 3, the gender pay differential was further attenuated to 32 cents (t = 6.7, p < .0001). The remaining 32 cent difference (56.6%) in earnings is inferred to be attributable to gender differences in HIT completion speed.

### Task selection analyses

Although completion speed appears to account for a significant portion of the pay gap, of particular interest are gender differences in task selection. Beyond structural factors such as education, household composition and completion speed, task selection accounts for a meaningful portion of the gender pay gap. As a reminder, the pay rate and expected completion time are posted for every HIT, so why women would select less remunerative tasks on average than men do is an important question to explore. In the next section of the paper we perform a set of analyses to examine factors that could account for this observed gender difference in task selection.

#### Advertised hourly pay

To examine gender differences in task selection, we used linear regression to directly examine whether the advertised hourly pay differed for tasks accepted by male and female workers. We first ran a simple model (Table 3; Model 3A) on the full dataset of 4.93 million HITs, with gender as the predictor and advertised hourly pay as the outcome including no other covariates. The unadjusted regression results (Model 4) shown in Table 3, indicates that, summed across all clusters and demographic groups, tasks completed by women were advertised as paying 28 cents (95% CI: $0.25-$0.31) less per hour (5.8%) compared to tasks completed by men (t = 21.8, p < .0001).

**Table 3:** Unadjusted and adjusted linear regression of *advertised* hourly pay on gender (N = 4,936,207).

**Advertised pay** **Model 4:** Predictor: Gender Adjustment: None	**β**	**SE**	**95% CI**	**p-value**

Women	-0.28	0.016	-0.34, -0.27	< .0001
**Model 5:** Predictor: Gender Adjustment: covariates*				
Women	-0.21	0.014	-0.23, -0.18	< .0001

*Models adjusted for race, marital status, number of children, and task clusters as categorical covariates, and age, HIT acceptance speed, and number of HITs as continuous covariates.

Model 5 examines whether the remuneration differences for tasks selected by men and women remains significant in the presence of multiple covariates included in the previous model and their interactions. The advertised pay differential for tasks selected by women compared to men was attenuated to 21 cents (4.3%), and remained statistically significant (t = 9.9, p < .0001). This estimate closely corresponded to the inferred influence of task selection reported in Table 2. Tests of gender by covariate interactions were significant only in the cases of age and marital status; the pay differential in tasks selected by men and women decreased with age and was more pronounced among single versus currently or previously married women.

To further examine what factors may account for the observed gender differences in task selection we plotted the observed pay gap within demographic and other covariate groups. Table 4 shows the distribution of tasks completed by men and women, as well as mean earnings and the pay gap across all demographic groups, based on the *advertised* (not actual) hourly pay for HITs selected (hereafter referred to as “advertised hourly pay” and the “advertised pay gap”). The average task was advertised to pay $4.88 per hour (95% CI $4.69, $5.10).

**Table 4:** Characteristics of MTurk workers in the CloudResearch database and the distribution of HITs, average advertised hourly pay, and gender pay gaps by individual-level subgroups.

	Total HITs		Mean HITs per Worker		Mean Advertised Hourly Pay		Mean Gender Gap in Advertised Hourly Pay
	Male	Female	Male	Female	Male	Female	
**TOTAL**	N = 2,396,978 (48.6%)	N = 2,539,229 (51.4%)	241	206	$4.87 CI: $4.86 - $4.87	$4.59 CI: $4.58 - $4.60	-$0.28 CI: -$0.25, -$0.31
**Age**							
18–29	733,449	602,078	203.28	165.77	$4.95 CI: $4.94 - $4.96	$4.63 CI: $4.62 - $4.64	-$0.31 CI: -$0.26. -$0.37
30–39	935,663	905,114	242.65	208.26	$4.93 CI: $4.92 - $4.94	$4.68 CI: $4.67 - $4.69	-$0.25 CI: -$0.20, -$0.31
40–49	399,718	456,955	269.90	217.29	$4.82 CI: $4.80 - $4.83	$4.55 CI: $4.54 - $4.57	-$0.26 CI: -$0.18, -$0.34
50–59	202,425	375,498	306.24	258.96	$4.65 CI: $4.64 - $4.67	$4.51 CI: $4.50 - $4.52	-$0.14 CI: -$0.04, -$0.24
60+	125,723	199,584	356.16	255.55	$4.30 CI: $4.28 - $4.31	$4.43 CI: $4.41 - $4.44	-$0.13 CI: $0.02, -$0.23
**Income**							
< 20k	645,605	694,642	232.73	207.73	$4.96 CI: $4.95 - $4.97	$4.67 CI: $4.66 - $4.68	-$0.28 CI: $0.22, -$0.35
20-39k	684,893	766,424	250.14	207.48	$4.90 CI: $4.89 - $4.91	$4.60 CI: $4.59 - $4.61	-$0.30 CI: -$0.24, -$0.36
40-59k	529,075	516,939	248.98	202.40	$4.84 CI: $4.83 - $4.85	$4.57 CI: $4.56 - $4.58	-$0.26 CI: -$0.20, -$0.33
60-79k	274,803	283,948	240.63	217.42	$4.78 CI: $4.76 - $4.79	$4.54 CI: $4.53 - $4.55	-$0.23 CI: -$0.16, -$0.31
80-99k	116,851	125,550	224.28	190.81	$4.71 CI: $4.69 - $4.73	$4.44 CI: $4.42 - $4.47	-$0.26 CI: -$0.14, -$0.39
100k+	145,751	151,726	211.54	200.70	$4.74 CI: $4.72 - $4.76	$4.47 CI: $4.46 - $4.49	-$0.27 CI: -$0.17, -$0.36
**Marital status**							
Never married	1,390,328	940,558	242.26	189.25	$4.97 CI: $4.96 - $4.97	$4.66 CI: $4.65 - $4.67	-$0.30 CI: -$0.25, -$0.35
Married	824,711	1,225,612	230.30	214.42	$4.74 CI: $4.73 - $4.75	$4.57 CI: $4.56 - $4.58	-$0.16 CI: -$0.11, -$0.21
Previously married	181,939	373,059	284.72	229.43	$4.70 CI: $4.69 - $4.72	$4.46 CI: $4.45 - $4.48	-$0.23 CI: -$0.13, -$0.34
**Children**							
0	1,583,991	1,129,463	237.34	195.07	$4.94 CI: $4.94 - $4.95	$4.68 CI: $4.67 - $4.69	-$0.26 CI: -$0.21, -$0.30
1–2	626,125	979,470	247.19	212.65	$4.74 CI: $4.73 - $4.75	$4.53 CI: $4.52 - $4.54	-$0.21 CI: -$0.15, -$0.27
3+	186,862	430,296	248.49	224.58	$4.67 CI: $4.66 - $4.69	$4.49 CI: $4.65-$4.50	-$0.18 CI: -$0.10, -$0.27
**Education**							
No College degree	1,262,163	1,405,325	245.65	214.32	$4.90 CI: $4.90 - $4.91	$4.59 CI: $4.59 - $4.60	-$0.31 CI: -$0.26, -$0.35
College degree	854,543	850,904	241.53	201.54	$4.87 CI: $4.87 - $4.88	$4.63 CI: $4.62 - $4.64	-$0.24 CI: -$0.19, -$0.29
Post-college degree	280,272	283,000	218.45	184.61	$4.69 CI: $4.68 - $4.71	$4.46 CI: $4.44 - $4.47	-$0.23 CI: -$0.15, -$0.31
**Race/ Ethnicity**							
White	1,830,078	1,981,698	244.50	207.51	$4.87 CI: $4.86 - $4.88	$4.59 CI: $4.58 - $5.00	-$0.28 CI: -$0.24, -$0.31
Asian	210,613	135,706	220.77	204.99	$4.93 CI: $4.91 - $4.95	$4.59 CI: $4.57 - $4.61	-$0.34 CI: -$0.21, -$0.47
Black	155,652	255,258	238.36	211.13	$4.78 CI: $4.76 - $4.80	$4.57 CI: $4.55 - $4.58	-$0.21 CI: -$0.10, -$0.32
Hispanic	165,820	116,016	235.54	195.64	$4.87 CI: $4.85 - $4.89	$4.68 CI: $4.66 - $4.70	-$0.19 CI: -$0.05, -$0.33

The pattern across demographic characteristics shows that the advertised hourly pay gap between genders is pervasive. Notably, a significant advertised gender pay gap is evident in every level of each covariate considered in Table 4, but more pronounced among some subgroups of workers. For example, the advertised pay gap was highest among the youngest workers ($0.31 per hour for workers age 18–29), and decreased linearly with age, declining to $0.13 per hour among workers age 60+. Advertised houry gender pay gaps were evident across all levels of education and income considered.

To further examine the potential influence of human capital factors on the advertised hourly pay gap, Table 5 presents the average advertised pay for selected tasks by level of experience on the CloudResearch platform. Workers were grouped into 4 experience levels, based on the number of prior HITs completed: Those who completed fewer than 100 HITs, between 100 and 500 HITs, between 500 and 1,000 HITs, and more than 1,000 HITs. A significant gender difference in advertised hourly pay was observed within each of these four experience groups. The advertised hourly pay for tasks selected by both male and female workers increased with experience, while the gender pay gap decreases. There was some evidence that male workers have more cumulative experience with the platform: 43% of male workers had the highest level of experience (previously completing 1,001–10,000 HITs) compared to only 33% of women.

**Table 5:** Experience level, cluster themes, and average advertised hourly pay for men and women.

	Analytic Sample		Total HITs		Mean No. of HITs		Mean Hourly Advertised Pay		Mean Gender Pay Gap
	Male	Female	Male	Female	Male	Female	Male	Female	
**Experience**									
0–100	9%	12%	280,198	404,357	62.01	61.27	$4.87 CI: $4.85 - $4.88	$4.61 CI: $4.59 - $4.62	-$0.27 CI: -$0.24, -$0.30
101–500	27%	33%	816,473	1,074,898	284.33	277.86	$5.13 CI: $5.12 - $5.14	$4.82 CI: $4.81 - $4.83	-$0.31 CI: -$0.28, -$0.34
501–1000	21%	21%	645,805	699,215	716.01	719.46	$5.32 CI: $5.31 - $5.34	$5.07 CI: $5.06 - $5.08	-$0.25 CI: -$0.19, -$0.31
1001–10000	43%	33%	1,301,602	1,077,372	1650.48	1513.35	$5.34 CI: $5.33 - $5.35	$5.16 CI: $5.15 - $5.17	-$0.18 CI: -$0.12, -$0.24
**Task Clusters**									
Evaluating, Rating, Perceptions	27.50%	28.19%	804,730	872,473	1144.1	893.08	$4.97 CI: $4.96 - $4.97	$4.62 CI: $4.61 - $4.62	-$0.35 CI: -$0.32, -$0.38
Short surveys which mention time duration	4.04%	3.88%	118,114	120,061	1127.94	918.04	$5.37 CI: $5.36 - $5.38	$5.17 CI: $5.16 - $5.19	-$0.20 CI: -$0.17, -$0.22
Academic, research studies	12.85%	12.51%	376,102	387,022	1177.98	938.72	$5.47 CI: $5.46 - $5.49	$5.23 CI: $5.21 - $5.24	-$0.25 CI: -$0.21, -$0.28
Surveys about attitudes and beliefs, opinions and experiences	1.68%	1.65%	49,084	50,914	1068.29	841.51	$5.74 CI: $5.71 - $5.76	$5.48 CI: $5.53 - $5.57	-$0.26 CI: -$0.30, -$0.22
Consumer surveys, purchases, behaviors, marketing	21.37%	21.66%	625,585	670,137	1122.11	882.55	$5.18 CI: $5.17 - $5.19	$4.91 CI: $4.90 - $4.92	-$0.27 CI: $0.24, -$0.30
Social attitudes	3.73%	4.05%	109,234	125,394	1060.93	805.97	$4.16 CI: $4.15 - $4.18	$3.86 CI: $3.85 - $3.87	-$0.30 CI: -$0.27, -$0.34
Games	1.73%	1.67%	50,640	51,790	1110.96	886.83	$5.55 CI: $5.52 - $5.59	$5.25 CI: $5.22 - $5.28	-$0.30 CI: -$0.25, -$0.36
"Answer a survey about…"	3.28%	3.37%	95,960	104,411	1088.95	860.83	$4.77 CI: $4.76 - $4.78	$4.63 CI: $4.61–4.64	-$0.15 CI: -$0.12, -$0.17
Decision making	6.20%	5.81%	181,448	179,731	1174.91	951.17	$5.33 CI: $5.32 - $5.34	$5.18 CI: $5.17 - $5.19	-$0.15 CI: -$0.12, -$0.18
“Short survey”	7.81%	7.58%	228,640	234,674	1131.87	897.98	$5.63 CI: $5.62 - $5.64	$5.52 CI: $5.51 - $5.53	-$0.11 CI: -$0.09, -$0.14
“Short study“	2.27%	2.33%	66,428	72,243	1120.43	874.02	$5.59 CI: $5.55 - $5.63	$5.23 CI: $5.20 - $5.27	-$0.36 CI: -$0.29, -$0.42
Psychology studies	1.70%	1.76%	49,711	54,424	1135.55	903.52	$4.80 CI: $4.78 - $4.82	$4.60 CI: $4.58 - $4.62	-$0.20 CI: -$0.15, -$0.25

Table 5 also explores the influence of task heterogeneity upon HIT selection and the gender gap in advertised hourly pay. K-means clustering was used to group HITs into 20 clusters initially based on the presence or absence of 5,140 distinct words appearing in HIT titles. Clusters with fewer than 50,000 completed tasks were then excluded from analysis. This resulted in 13 clusters which accounted for 94.3% of submitted work assignments (HITs).

The themes of all clusters as well as the average hourly advertised pay for men and women within each cluster are presented in the second panel of Table 5. The clusters included categories such as Games, Decision making, Product evaluation, Psychology studies, and Short Surveys. We did not observe a gender preference for any of the clusters. Specifically, for every cluster, the proportion of males was no smaller than 46.6% (consistent with the slightly lower proportion of males on the platform, see Table 1) and no larger than 50.2%. As shown in Table 5, the gender pay gap was observed within each of the clusters. These results suggest that residual task heterogeneity, a proxy for occupational segregation, is not likely to contribute to a gender pay gap in this market.

Task length was defined as the advertised estimated duration of a HIT. Table 6 presents the advertised hourly gender pay gaps for five categories of HIT length, which ranged from a few minutes to over 1 hour. Again, a significant advertised hourly gender pay gap was observed in each category.

**Table 6:** Distribution of HITs, average hourly advertised pay, and gender gaps in advertised hourly pay by advertised HIT duration.

	Analytic Sample		Total HITs		Mean No. of HITs		Mean Hourly Advertised Pay		Mean Gender Pay Gap
Advertised Duration (minutes)	Male	Female	Male	Female	Male	Female	Male	Female	
0–5	24%	23%	580,969	595,793	752.17	617.50	$6.77 CI: $6.75–6.79	$6.47 CI: $6.45 - $6.49	-$0.29 CI: -$0.25, -$0.35
5–10	32%	30%	761,543	772,963	798.10	655.79	$5.23 CI: $5.22 - $5.23	$5.06 CI: $5.06 - $5.06	-$0.17 CI: -$0.14, -$0.19
10–30	38%	39%	908,853	991,595	805.00	645.52	$4.51 CI: $4.50 - $4.51	$4.25 CI: $4.24.—$4.25	-$0.26 CI: -$0.22, -$0.30
30–60	5%	6%	126,051	156,033	775.28	610.07	$3.55 CI: $3.54 - $ 3.56	$3.21 CI: $3.20 - $3.23	-$0.33 CI: -$0.28, -$0.39
60+	1%	1%	19,562	22,845	822.89	655.63	$3.75 CI: $3.71 - $3.79	$3.34 CI: $3.31 - $3.38	-$0.40 CI: -$0.31, -$0.50

Finally, we conducted additional supplementary analyses to determine if other plausible factors such as HIT timing could account for the gender pay gap. We explored temporal factors including hour of the day and day of the week. Each completed task was grouped based on the hour and day in which it was completed. A significant advertised gender pay gap was observed within each of the 24 hours of the day and for every day of the week demonstrating that HIT timing could not account for the observed gender gap (results available in Supplementary Materials).

## Discussion

In this study we examined the gender pay gap on an anonymous online platform across an 18-month period, during which close to five million tasks were completed by over 20,000 unique workers. Due to factors that are unique to the Mechanical Turk online marketplace–such as anonymity, self-selection into tasks, relative homogeneity of the tasks performed, and flexible work scheduling–we did not expect earnings to differ by gender on this platform. However, contrary to our expectations, a robust and persistent gender pay gap was observed.

The average estimated actual pay on MTurk over the course of the examined time period was $5.70 per hour, with the gender pay differential being 10.5%. Importantly, gig economy platforms differ from more traditional labor markets in that hourly pay largely depends on the speed with which tasks are completed. For this reason, an analysis of gender differences in actual earned pay will be affected by gender differences in task completion speed. Unfortunately, we were not able to directly measure the speed with which workers complete tasks and account for this factor in our analysis. This is because workers have the ability to accept multiple HITs at the same time and multiple HITs can sit dormant in a queue, waiting for workers to begin to work on them. Therefore, the actual time that many workers spend working on tasks is likely less than what is indicated in the metadata available. For this reason, the estimated average actual hourly rate of $5.70 is likely an underestimate and the gender gap in actual pay cannot be precisely measured. We infer however, by the residual gender pay gap after accounting for other factors, that as much as 57% (or $.32) of the pay differential may be attributable to task completion speed. There are multiple plausible explanations for gender differences in task completion speed. For example, women may be more meticulous at performing tasks and, thus, may take longer at completing them. There may also be a skill factor related to men’s greater experience on the platform (see Table 5), such that men may be faster on average at completing tasks than women.

However, our findings also revealed another component of a gender pay gap on this platform–gender differences in the selection of tasks based on their advertised pay. Because the speed with which workers complete tasks does not impact these estimates, we conducted extensive analyses to try to explain this gender gap and the reasons why women appear on average to be selecting tasks that pay less compared to men. These results pertaining to the *advertised* gender pay gap constitute the main focus of this study and the discussion that follows.

The overall advertised hourly pay was $4.88. The gender pay gap in the advertised hourly pay was $0.28, or 5.8% of the advertised pay. Once a gender earnings differential was observed based on advertised pay, we expected to fully explain it by controlling for key structural and individual-level covariates. The covariates that we examined included experience, age, income, education, family composition, race, number of children, task length, the speed of accepting a task, and thirteen types of subtasks. We additionally examined the time of day and day of the week as potential explanatory factors. Again, contrary to our expectations, we observed that the pay gap persisted even after these potential confounders were controlled for. Indeed, separate analyses that examined the advertised pay gap within each subcategory of the covariates showed that the pay gap is ubiquitous, and persisted within each of the ninety sub-groups examined. These findings allows us to rule out multiple mechanisms that are known drivers of the pay gap in traditional labor markets and other gig economy marketplaces. To our knowledge this is the only study that has observed a pay gap across such diverse categories of workers and conditions, in an anonymous marketplace, while simultaneously controlling for virtually all variables that are traditionally implicated as causes of the gender pay gap.

### Individual-level factors

Individual-level factors such as parental status and family composition are a common source of the gender pay gap in traditional labor markets *[15]*. Single mothers have previously been shown to have lower reservation wages compared to other men and women [21]. In traditional labor markets lower reservation wages lead single mothers to be willing to accept lower-paying work, contributing to a larger gender pay gap in this group. This pattern may extend to gig economy markets, in which single mothers may look to online labor markets as a source of supplementary income to help take care of their children, potentially leading them to become less discriminating in their choice of tasks and more willing to work for lower pay. Since female MTurk workers are 20% more likely than men to have children (see Table 1), it was critical to examine whether the gender pay gap may be driven by factors associated with family composition.

An examination of the advertised gender pay gap among individuals who differed in their marital and parental status showed that while married workers and those with children are indeed willing to work for lower pay (suggesting that family circumstances do affect reservation wages and may thus affect the willingness of online workers to accept lower-paying online tasks), women’s hourly pay is consistently lower than men’s within both single and married subgroups of workers, and among workers who do and do not have children. Indeed, contrary to expectations, the advertised gender pay gap was highest among those workers who are single, and among those who do not have any children. This observation shows that it is not possible for parental and family status to account for the observed pay gap in the present study, since it is precisely among unmarried individuals and those without children that the largest pay gap is observed.

Age was another factor that we considered to potentially explain the gender pay gap. In the present sample, the hourly pay of older individuals is substantially lower than that of younger workers; and women on the platform are five years older on average compared to men (see Table 1). However, having examined the gender pay gap separately within five different age cohorts we found that the largest pay gap occurs in the two youngest cohort groups: those between 18 and 29, and between 30 and 39 years of age. These are also the largest cohorts, responsible for 64% of completed work in total.

Younger workers are also most likely to have never been married or to not have any children. Thus, taken together, the results of the subgroup analyses are consistent in showing that the largest pay gap does not emerge from factors relating to parental, family, or age-related person-level factors. Similar patterns were found for race, education, and income. Specifically, a significant gender pay gap was observed within each subgroup of every one of these variables, showing that person-level factors relating to demographics are not driving the pay gap on this platform.

### Experience

Experience is a factor that has an influence on the pay gap in both traditional and gig economy labor markets *[20]*. As noted above, experienced workers may be faster and more efficient at completing tasks in this platform, but also potentially more savvy at selecting more remunerative tasks compared to less experienced workers if, for example, they are better at selecting tasks that will take less time to complete than estimated on the dashboard [20]. On MTurk, men are overall more experienced than women. However, experience does not account for the gender gap in advertised pay in the present study. Inexperienced workers comprise the vast majority of the Mechanical Turk workforce, accounting for 67% of all completed tasks (see Table 5). Yet within this inexperienced group, there is a consistent male earning advantage based on the advertised pay for tasks performed. Further, controlling for the effect of experience in our models has a minimal effect on attenuating the gender pay gap.

### Task heterogeneity

Another important source of the gender pay gap in both traditional and gig economy labor markets is task heterogeneity. In traditional labor markets men are disproportionately represented in lucrative fields, such as those in the tech sector [23]. While the workspace within MTurk is relatively homogeneous compared to the traditional labor market, there is still some variety in the kinds of tasks that are available, and men and women may have been expected to have preferences that influence choices among these.

To examine whether there is a gender preference for specific tasks, we systematically analyzed the textual descriptions of all tasks included in this study. These textual descriptions were available for all workers to examine on their dashboards, along with information about pay. The clustering algorithm revealed thirteen categories of tasks such as games, decision making, several different kinds of survey tasks, and psychology studies.We did not observe any evidence of gender preference for any of the task types. Within each of the thirteen clusters the distribution of tasks was approximately equally split between men and women. Thus, there is no evidence that women as a group have an overall preference for specific tasks compared to men. Critically, the gender pay gap was also observed within each one of these thirteen clusters.

Another potential source of heterogeneity is task length. Based on traditional labor markets, one plausible hypothesis about what may drive women’s preferences for specific tasks is that women may select tasks that differ in their duration. For example, women may be more likely to use the platform for supplemental income, while men may be more likely to work on HITs as their primary income source. Women may thus select shorter tasks relative to their male counterparts. If the shorter tasks pay less money, this would result in what appears to be a gender pay gap.

However, we did not observe gender differences in task selection based on task duration. For example, having divided tasks into their advertised length, the tasks are preferred equally by men and women. Furthermore, the shorter tasks’ hourly pay is substantially higher on average compared to longer tasks.

Additional evidence that scheduling factors do not drive the gender pay gap is that it was observed within all hourly and daily intervals (See S1 and S2 Tables in Appendix). These data are consistent with the results presented above regarding personal level factors, showing that the majority of male and female Mechanical Turk workers are single, young, and have no children. Thus, while in traditional labor markets task heterogeneity and labor segmentation is often driven by family and other life circumstances, the cohort examined in this study does not appear to be affected by these factors.

### Practical implications of a gender pay gap on online platforms for social and behavioral science research

The present findings have important implications for online participant recruitment in the social and behavioral sciences, and also have theoretical implications for understanding the mechanisms that give rise to the gender pay gap. The last ten years have seen a revolution in data collection practices in the social and behavioral sciences, as laboratory-based data collection has slowly and steadily been moving online [16, 24]. Mechanical Turk is by far the most widely used source of human participants online, with thousands of published peer-reviewed papers utilizing Mechanical Turk to recruit at least some of their human participants [25]. The present findings suggest both a challenge and an opportunity for researchers utilizing online platforms for participant recruitment. Our findings clearly reveal for the first time that sampling research participants on anonymous online platforms tends to produce gender pay inequities, and that this happens independent of demographics or type of task. While it is not clear from our findings what the exact cause of this inequity is, what is clear is that the online sampling environment produces similar gender pay inequities as those observed in other more traditional labor markets, after controlling for relevant covariates.

This finding is inherently surprising since many mechanisms that are known to produce the gender pay gap in traditional labor markets are not at play in online microtasks environments. Regardless of what the generative mechanisms of the gender pay gap on online microtask platforms might be, researchers may wish to consider whether changes in their sampling practices may produce more equitable pay outcomes. Unlike traditional labor markets, online data collection platforms have built-in tools that can allow researchers to easily fix gender pay inequities. Researchers can simply utilize gender quotas, for example, to fix the ratio of male and female participants that they recruit. These simple fixes in sampling practices will not only produce more equitable pay outcomes but are also most likely advantageous for reducing sampling bias due to gender being correlated with pay. Thus, while our results point to a ubiquitous discrepancy in pay between men and women on online microtask platforms, such inequities have relatively easy fixes on online gig economy marketplaces such as MTurk, compared to traditional labor markets where gender-based pay inequities have often remained intractable.

### Other gig economy markets

As discussed in the introduction, a gender wage gap has been demonstrated on Uber, a gig economy transportation marketplace [20], where men earn approximately 7% more than women. However, unlike in the present study, the gender wage gap on Uber was fully explained by three factors; a) driving speed predicted higher wages, with men driving faster than women, b) men were more likely than women to drive in congested locations which resulted in better pay, c) experience working for Uber predicted higher wages, with men being more experienced. Thus, contrary to our findings, the gender wage gap in gig economy markets studied thus far are fully explained by task heterogeneity, experience, and task completion speed. To our knowledge, the results presented in the present study are the first to show that the gender wage gap can emerge independent of these factors.

### Generalizability

Every labor market is characterized by a unique population of workers that are almost by definition not a representation of the general population outside of that labor market. Likewise, Mechanical Turk is characterized by a unique population of workers that is known to differ from the general population in several ways. Mechanical Turk workers are younger, better educated, less likely to be married or have children, less likely to be religious, and more likely to have a lower income compared to the general United States population [24]. The goal of the present study was not to uncover universal mechanisms that generate the gender pay gap across all labor markets and demographic groups. Rather, the goal was to examine a highly unique labor environment, characterized by factors that should make this labor market immune to the emergence of a gender pay gap.

Previous theories accounting for the pay gap have identified specific generating mechanisms relating to structural and personal factors, in addition to discrimination, as playing a role in the emergence of the gender pay gap. This study examined the work of over 20,000 individuals completing over 5 million tasks, under conditions where standard mechanisms that generate the gender pay gap have been controlled for. Nevertheless, a gender pay gap emerged in this environment, which cannot be accounted for by structural factors, demographic background, task preferences, or discrimination. Thus, these results reveal that the gender pay gap can emerge—in at least some labor markets—in which discrimination is absent and other key factors are accounted for. These results show that factors which have been identified to date as giving rise to the gender pay gap are not sufficient to explain the pay gap in at least some labor markets.

### Potential mechanisms

While we cannot know from the results of this study what the actual mechanism is that generates the gender pay gap on online platforms, we suggest that it may be coming from outside of the platform. The particular characteristics of this labor market—such as anonymity, relative task homogeneity, and flexibility—suggest that, everything else being equal, women working in this platform have a greater propensity to choose less remunerative opportunities relative to men. It may be that these choices are driven by women having a lower reservation wage compared to men [21, 26]. Previous research among student populations and in traditional labor markets has shown that women report lower pay or reward expectations than men [27–29]. Lower pay expectations among women are attributed to justifiable anticipation of differential returns to labor due to factors such as gender discrimination and/or a systematic psychological bias toward pessimism relative to an overly optimistic propensity among men [30].

Our results show that even if the bias of employers is removed by hiding the gender of workers as happens on MTurk, it seems that women may select lower paying opportunities themselves because their lower reservation wage influences the types of tasks they are willing to work on. It may be that women do this because cumulative experiences of pervasive discrimination lead women to undervalue their labor. In turn, women’s experiences with earning lower pay compared to men on traditional labor markets may lower women’s pay expectations on gig economy markets. Thus, consistent with these lowered expectations, women lower their reservation wages and may thus be more likely than men to settle for lower paying tasks.

More broadly, gender norms, psychological attributes, and non-cognitive skills, have recently become the subject of investigation as a potential source for the gender pay gap [3], and the present findings indicate the importance of such mechanisms being further explored, particularly in the context of task selection. More research will be required to explore the potential psychological and antecedent structural mechanisms underlying differential task selection and expectations of compensation for time spent on microtask platforms, with potential relevance to the gender pay gap in traditional labor markets as well. What these results do show is that pay discrepancies can emerge despite the absence of discrimination in at least some circumstances. These results should be of particular interest for researchers who may wish to see a more equitable online labor market for academic research, and also suggest that novel and heretofore unexplored mechanisms may be at play in generating these pay discrepancies.

A final note about framing: we are aware that explanations of the gender pay gap that invoke elements of women’s agency and, more specifically, “choices” risk both; a) diminishing or distracting from important structural factors, and b) “naturalizing” the status quo of gender inequality *[30]*. As Connor and Fiske (2019) argue, causal attributions for the gender pay gap to “unconstrained choices” by women, common as part of human capital explanations, may have the effect, intended or otherwise, of reinforcing system-justifying ideologies that serve to perpetuate inequality. By explicitly locating women’s economic decision making on the MTurk platform in the broader context of inegalitarian gender norms and labor market experiences outside of it (as above), we seek to distance our interpretation of our findings from implicit endorsement of traditional gender roles and economic arrangements and to promote further investigation of how the observed gender pay gap in this niche of the gig economy may reflect both broader gender inequalities and opportunities for structural remedies.

## Supporting information

S1 Table Distribution of HITs, average pays, and gender pay gaps by hour of day. (PDF)

S2 Table Distribution of HITs, average pays, and gender pay gaps by day of the week. (PDF)
