# Supplementary material for: The persistence of pay inequality: The gender pay gap in an anonymous online labor market
Source: PLoS One. 2020 Feb 21;15(2):e0229383. doi: 10.1371/journal.pone.0229383 (PMC7034870; doi:10.1371/journal.pone.0229383)
Supplement: S1 Table — (PDF) [file pone.0229383.s001.pdf]

Table S1. Distribution of HITs, average pay, and gender pay gaps by hour of day.

	Analytic Sample		Total HITs		Mean No. of HITs		Mean Hourly Pay		Mean Gender Pay Gap
Hour of the day	Males	Females	Male	Female	Male	Female	Male	Female	
0:00	4%	4%	97,018	109,199	753.38	601.07	$4.66<br>CI: $4.64 - $4.68	$4.36<br>CI: $4.34 - $4.38	-$0.30<br>CI:- $0.35, -$0.26
1:00	4%	4%	85,993	101,498	717.74	577.58	$4.60<br>CI: $4.57 - $4.62	$4.27<br>CI: $4.25 - $4.30	-$0.32<br>CI: -$0.37, -$0.27
2:00	3%	4%	75,178	90,618	703.70	558.97	$4.58<br>CI: $4.55 - $4.61	$4.23<br>CI: $4.20 - $4.25	-$0.35<br>CI: -$0.41, -$0.29
3:00	3%	3%	65,334	75,461	710.19	545.92	$4.46<br>CI: $4.43 - $4.48	$4.13<br>CI: $4.11 - $4.16	-$0.32<br>CI: -$0.37, -$0.27
4:00	2%	2%	54,644	59,849	694.36	531.20	$4.44<br>CI: $4.42 - $4.47	$4.15<br>CI: $4.13 - $4.18	-$0.29<br>CI: -$0.35, -$0.24
5:00	2%	2%	43,909	45,032	669.55	522.68	$4.54<br>CI: $4.40 - $4.57	$4.19<br>CI: $4.16 - $4.22	-$0.34<br>CI: -$0.41, -$0.27
6:00	2%	1%	36,822	35,600	660.90	520.90	$4.49<br>CI: $4.46 - $4.53	$4.27<br>CI: $ 4.23 - $4.31	-$0.22<br>CI: -$0.29, -$0.15
7:00	1%	1%	31,849	27,459	704.17	558.65	$4.64<br>CI: $4.59 - $4.68	$4.41<br>CI: $4.36 - $4.45	-$0.23<br>CI: -$0.31, -$0.14
8:00	1%	0.97%	27,883	24,567	748.82	651.91	$4.60<br>CI: $4.56 - $4.65	$4.36<br>CI: $4.31 - $4.41	-$0.24<br>CI: -$0.33, -$0.15
9:00	1%	1%	27,446	26,355	789.01	661.90	$4.65<br>CI: $4.61 - $4.69	$4.41<br>CI: $4.37 - $4.45	-$0.24<br>CI: -$0.32, -$0.15
10:00	1%	1%	33,889	34,847	796.84	656.17	$4.59<br>CI: $4.55 - $4.64	$4.40<br>CI: $4.36 - $4.43	-$0.20<br>CI: -$0.28, -$0.11
11:00	2%	2%	47,231	50,267	829.85	637.07	$4.58<br>CI: $4.55 - $4.61	$4.36<br>CI: $4.34 - $4.39	-$0.21<br>CI: -$0.29, -$0.14
12:00	3%	3%	68,838	77,195	830.54	657.16	$4.70<br>CI: $4.67 - $4.73	$4.47<br>CI: $4.44 - $4.50	-$0.23<br>CI: -$0.30, -$0.16
13:00	5%	5%	109,803	123,465	812.08	670.11	$4.89<br>CI: $4.86 - $4.91	$4.63<br>CI: $4.61 - $4.65	-$0.25<br>CI: -$0.30, -$0.20
14:00	6%	7%	153,030	166,355	809.34	685.85	$5.06<br>CI: $5.04 - $5.09	$4.78<br>CI: $4.76 - $4.80	-$0.28<br>CI: -$0.33, -$0.23
15:00	8%	8%	181,213	194,303	810.51	686.82	$5.04<br>CI: $5.02 - $5.06	$4.80<br>CI: $4.78 - $4.82	-$0.24<br>CI: -$0.28, -$0.19
16:00	8%	7%	181,807	188,872	818.89	679.28	$4.98<br>CI: $4.96 - $4.99	$4.74<br>CI: $4.72 - $4.75	-$0.24<br>CI: -$0.28, -$0.20
17:00	8%	7%	183,528	188,933	820.53	666.76	$5.06<br>CI: $5.04 - $5.08	$4.77<br>CI: $4.75 - $4.79	-$0.29<br>CI: -$0.33, -$0.24
18:00	7%	7%	179,404	185,721	813.21	653.50	$5.04<br>CI: $5.02 - $5.06	$4.76<br>CI: $4.74 - $4.77	-$0.28<br>CI: -$0.32, -$0.24
19:00	7%	7%	171,712	175,840	810.18	656.62	$4.97<br>CI: $4.96 - $4.99	$4.72<br>CI: $4.71 - $4.74	-$0.25<br>CI: -$0.29, -$0.21
20:00	7%	6%	161,169	163,246	811.01	648.72	$4.94<br>CI: $4.92 - $4.96	$4.68<br>CI: $4.66 - $4.70	-$0.26<br>CI: -$0.30, -$0.22
21:00	6%	6%	144,797	146,183	800.87	644.71	$5.05<br>CI: $5.03 - $5.07	$4.76<br>CI: $4.74 - $4.78	-$0.30<br>CI: -$0.34, -$0.25
22:00	5%	5%	124,614	129,238	789.67	639.86	$4.97<br>CI: $4.94 - $4.99	$4.67<br>CI: $4.64 - $4.69	-$0.30<br>CI: -$0.35, -$0.25
23:00	5%	5%	109,867	119,126	777.50	625.67	$4.80<br>CI: $4.78 - $4.82	$4.49<br>CI: $4.47 - $4.51	-$0.31<br>CI: -$0.35, -$0.26
