# Supplementary material for: The persistence of pay inequality: The gender pay gap in an anonymous online labor market
Source: PLoS One. 2020 Feb 21;15(2):e0229383. doi: 10.1371/journal.pone.0229383 (PMC7034870; doi:10.1371/journal.pone.0229383)
Supplement: S2 Table — (PDF) [file pone.0229383.s002.pdf]

Table S2. Distribution of HITs, average pay, and gender pay gaps by day of the week.

	Analytic Sample		Total HITs		Mean No. of HITs		Mean Hourly Pay		Mean Gender Pay Gap
Day of the week	Males	Females	Male	Female	Male	Female	Male	Female	
Sunday	7%	7%	162,362	189,156	711.17	563.50	$4.47<br>CI: $4.45 - $4.49	$4.17<br>CI: 4.15 - $4.19	-$0.30<br>CI: -$0.34, -$0.26
Monday	16%	17%	379,111	434,843	797.14	645.95	$4.94<br>CI: $4.93 - $4.95	$4.65<br>CI: $4.64 - $4.66	-$0.28<br>CI: -$0.32, -$0.25
Tuesday	18%	17%	434,843	449,580	798.07	652.31	$4.96<br>CI: $4.95 - $4.97	$4.71<br>CI: $4.70 - $4.72	-$0.26<br>CI: -$0.29, -$0.22
Wednesday	18%	17%	425,397	446,540	796.83	651.40	$5.00<br>CI: $4.98 - $5.01	$4.72<br>CI: $4.71 - $4.73	-$0.27<br>CI: -$0.31, -$0.24
Thursday	18%	17%	420,889	437,647	808.71	660.00	$4.93<br>CI: $4.92 - $4.95	$4.69<br>CI: $4.68 - $4.70	-$0.25<br>CI: -$0.28, -$0.21
Friday	15%	15%	363,006	379,593	799.63	649.10	$4.84<br>CI: $4.83 - $4.86	$4.56<br>CI: $4.54 - $4.57	-$0.29<br>CI: -$0.32, -$0.25
Saturday	9%	9%	211,370	243,095	737.66	594.19	$4.56<br>CI: $4.54 - $4.57	$4.27<br>CI: $4.26 - $4.29	-$0.28<br>CI: -$0.32, -$0.25
